# Hyper-accumulation of starch and oil in a *Chlamydomonas* mutant affected in a plant-specific DYRK kinase

**DOI:** 10.1186/s13068-016-0469-2

**Published:** 2016-03-08

**Authors:** Miriam Schulz-Raffelt, Vincent Chochois, Pascaline Auroy, Stéphan Cuiné, Emmanuelle Billon, David Dauvillée, Yonghua Li-Beisson, Gilles Peltier

**Affiliations:** CEA, CNRS, Aix-Marseille Université, Institut de Biosciences et Biotechnologies Aix Marseille, Laboratoire de Bioénergétique et Biotechnologie des Bactéries et Microalgues, CEA Cadarache, 13108 Saint-Paul-lez-Durance, France; CNRS, Biologie Végétale et Microbiologie Environnementale, UMR7265, 13108 Saint-Paul-lez-Durance, France; Aix Marseille Université, Biologie Végétale et Microbiologie Environnementale, UMR7265, 13284 Marseille, France; UMR8576, CNRS, Université des Sciences et Technologies de Lille, 59655 Villeneuve d’Ascq, France; Molecular Biotechnology and Systems Biology, TU Kaiserslautern, Paul-Ehrlich-Straße 23, 67663 Kaiserslautern, Germany; Research School of Biology College of Medicine, Biology and Environment, Linneaus Building 134, The Australian National University, Canberra, ACT 2601 Australia

**Keywords:** *Chlamydomonas*, DYRK, Kinase, Microalgae, Nutrient deprivation, Oil, Photosynthesis, Starch

## Abstract

**Background:**

Because of their high biomass productivity and their ability to accumulate high levels of energy-rich reserve compounds such as oils or starch, microalgae represent a promising feedstock for the production of biofuel. Accumulation of reserve compounds takes place when microalgae face adverse situations such as nutrient shortage, conditions which also provoke a stop in cell division, and down-regulation of photosynthesis. Despite growing interest in microalgal biofuels, little is known about molecular mechanisms controlling carbon reserve formation. In order to discover new regulatory mechanisms, and identify genes of interest to boost the potential of microalgae for biofuel production, we developed a forward genetic approach in the model microalga *Chlamydomonas reinhardtii*.

**Results:**

By screening an insertional mutant library on the ability of mutants to accumulate and re-mobilize reserve compounds, we isolated a *Chlamydomonas* mutant (*starch degradation 1, std1*) deficient for a dual-specificity tyrosine-phosphorylation-regulated kinase (DYRK). The *std1* mutant accumulates higher levels of starch and oil than wild-type and maintains a higher photosynthetic activity under nitrogen starvation. Phylogenetic analysis revealed that this kinase (named DYRKP) belongs to a plant-specific subgroup of the evolutionarily conserved DYRK kinase family. Furthermore, hyper-accumulation of storage compounds occurs in *std1* mostly under low light in photoautotrophic condition, suggesting that the kinase normally acts under conditions of low energy status to limit reserve accumulation.

**Conclusions:**

The DYRKP kinase is proposed to act as a negative regulator of the sink capacity of photosynthetic cells that integrates nutrient and energy signals. Inactivation of the kinase strongly boosts accumulation of reserve compounds under photoautotrophic nitrogen deprivation and allows maintaining high photosynthetic activity. The DYRKP kinase therefore represents an attractive target for improving the energy density of microalgae or crop plants.

**Electronic supplementary material:**

The online version of this article (doi:10.1186/s13068-016-0469-2) contains supplementary material, which is available to authorized users.

## Background

Microalgae, the primary biomass producers of oceans, are a promising and renewable feedstock for the production of next-generation biofuels [1–4]. Despite their high biomass productivity and a marked ability to accumulate high intracellular amounts of energy-rich reserve compounds including starch [5] convertible into bioethanol and oil convertible into biodiesel [1, 6], algal productivity must be improved in order to implement economically viable biofuel production [7]. One of the major limitations is the requirement for stress conditions such as nutrient deprivation to trigger accumulation of reserve compounds, as this results in decreased biomass productivity [1]. In natural environments, photosynthetic organisms have developed sophisticated strategies to optimize growth and survival under constantly fluctuating conditions of light, temperature, or nutrient availability. Deprivation of essential macronutrients (such as nitrogen or sulfur) strongly affects algal growth and induces drastic changes in the cellular metabolism, including decreased protein synthesis, arrested cell division, a massive accumulation of energy-rich storage compounds [5, 8], and a down-regulation of photosynthesis [9–11]. Deciphering regulatory mechanisms that control photosynthesis and reserve accumulation in response to nutrient supply is therefore a key issue in understanding survival strategies of photosynthetic organisms in natural ecosystems, and is thus crucial for optimization of algal productivity for biotechnological applications.

Until now only a few regulatory elements and pathways linking the nutrient status to reserve accumulation and growth have been identified in plants. These include the conserved TOR and SnRK1/Snf1/AMPK kinases involved in the control of growth by nutrient availability [12, 13]. In *Chlamydomonas,* SNRK2 has been identified as a crucial regulatory element of the S deprivation response [14, 15]. A nitrogen response regulator (NRR1) predicted as a transcription factor and holding a SQUAMOSA promoter-binding domain was proposed to regulate the algal oil content [16]. In yeast, other regulatory elements such as the DYRK kinase (dual-specificity tyrosine-phosphorylation-regulated kinase) Yak1 are involved in the cellular response to nutrient stress [17]. Recently, a *Chlamydomonas* mutant deficient in a DYRK kinase belonging to the Yak1 subfamily was isolated from a forward genetic screen; this mutant is showing a decreased capacity to accumulate oil under N deprivation when compared to the wild-type [18].

With the aim to identify new regulatory mechanisms involved in the dynamics of reserve formation in response to nutrient availability, a genetic screen was developed in the unicellular green alga *Chlamydomonas reinhardtii* [19]. We report here the characterization of one such mutant, defected in a plant-specific DYRK kinase (DYRKP), which accumulates higher starch and oil amounts than WT in response to nutrient deprivation in photoautotrophic conditions.

## Results

### Molecular characterization and genetic complementation of the *std1* mutant

**Fig. 1:**
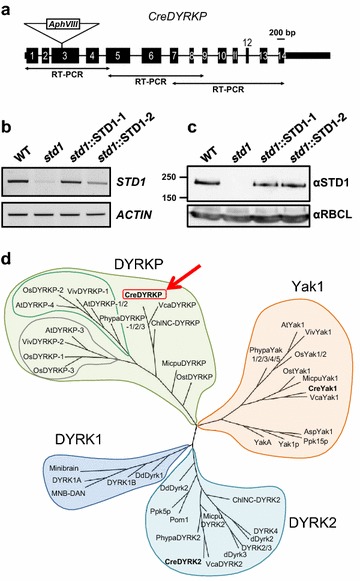
Molecular and phylogenetic characterization of the *Chlamydomonas* *std1* mutant. **a** In *std1*, the paromomycin-resistance cassette (*Aph*VIII gene, *white box*) is inserted within the third exon of a gene annotated as a DYRK kinase (Cre07.g337300, v5.5). The gene structure was deduced from three overlapping RT-PCR. **b** *STD1* transcript levels were analyzed by RT-PCR in WT, *std1* and two complemented lines (*std1*::STD1-1 and *std1*::STD1-2) by amplifying a 1421 bp product. A 456 bp RT-PCR actin product was amplified as a loading control. Sequence primers for RT-PCR are given in Additional file 1: Table S2. **c** The level of STD1 protein was analyzed by immunodetection in WT, *std1* and two complemented lines (*std1*::STD1-1 and *std1*::STD1-2). **d** Phylogenetic analysis of the DYRK protein family indicates that STD1 (referred to here as DYRKP) belongs to a new DYRK subfamily specific to plants

We previously initiated a genetic approach in the unicellular green alga *C. reinhardtii* by screening a DNA insertional mutant library based on the analysis of starch content dynamics. Insertion lines were submitted to 5 days N or S deprivation to induce starch accumulation, then to a 48-h starch degradation period under nutrient replete conditions. Eighteen mutants showing higher intracellular starch amounts than WT were isolated from the screen of 15,000 paromomycin-resistant transformants [19], among which one mutant, named *std1* for *st*arch *d*egradation *1*, contained a single insertion of the paromomycin (*Aph*VIII)-resistance cassette within the third exon of a gene, initially annotated as *DYRK2* (*C. reinhardtii* genome version 4.0) (Additional file 1: Figure S1A). A new gene structure was confirmed based on overlapping RT-PCRs (Fig. 1a), and the locus was renamed *DYRKP* based on the subsequent phylogenetic analysis. The *std1* mutant was complemented using a construct containing the wild-type *DYRKP* genomic sequence driven by the constitutive *psaD* promoter (Additional file 1: Figure S1B). Two independent complemented strains (*std1*::STD1-1 and *std1*::STD1-2) were isolated showing transcript and protein levels close to that of WT progenitor (Fig. 1b, c), and a rescued starch degradation phenotype (Additional file 1: Figure S1C).

### *Chlamydomonas* DYRKP (STD1) is a member of a novel plant-specific group of the DYRK family

DYRKs are a relatively novel subfamily of eukaryotic kinases belonging to the CMGC group, including CDKs (cyclin-dependent kinases) and MAPKs (mitogen-activated protein kinases). In the yeast *Saccharomyces cerevisiae*, the DYRK family member Yak1 is a negative regulator of cell proliferation under nutrition stress [20, 21]. DYRK kinases exhibit conserved sequence features, in particular the DYRK homology-box (DH-box) that precedes the conserved catalytic domain [22]. Phylogenetic analysis showed that the *Chlamydomonas* DYRKP (STD1) belongs to a distinct group, different from DYRK1, DYRK2, and Yak sub-families (Fig. 1d). This novel group contains only plant members and has been thus named DYRKP (for plant DYRK) [18]. DYRKP shares conserved sequence features of the other DYRK sub-families, including a DH-box motif (Additional file 1: Figure S2A, B). The length of the N- and C-terminal regions upstream and downstream of the kinase domain is quite variable among the DYRK family, but DYRKP members harbor a very short C-terminal extension (Additional file 1: Figure S2C). Whereas mosses and vascular plants harbor 2–6 DYRKP homologues, microalgal genomes tend to contain only one group member.

### The *std1* mutant hyper-accumulates starch and oil under photoautotrophic nutrient deprivation

**Fig. 2:**
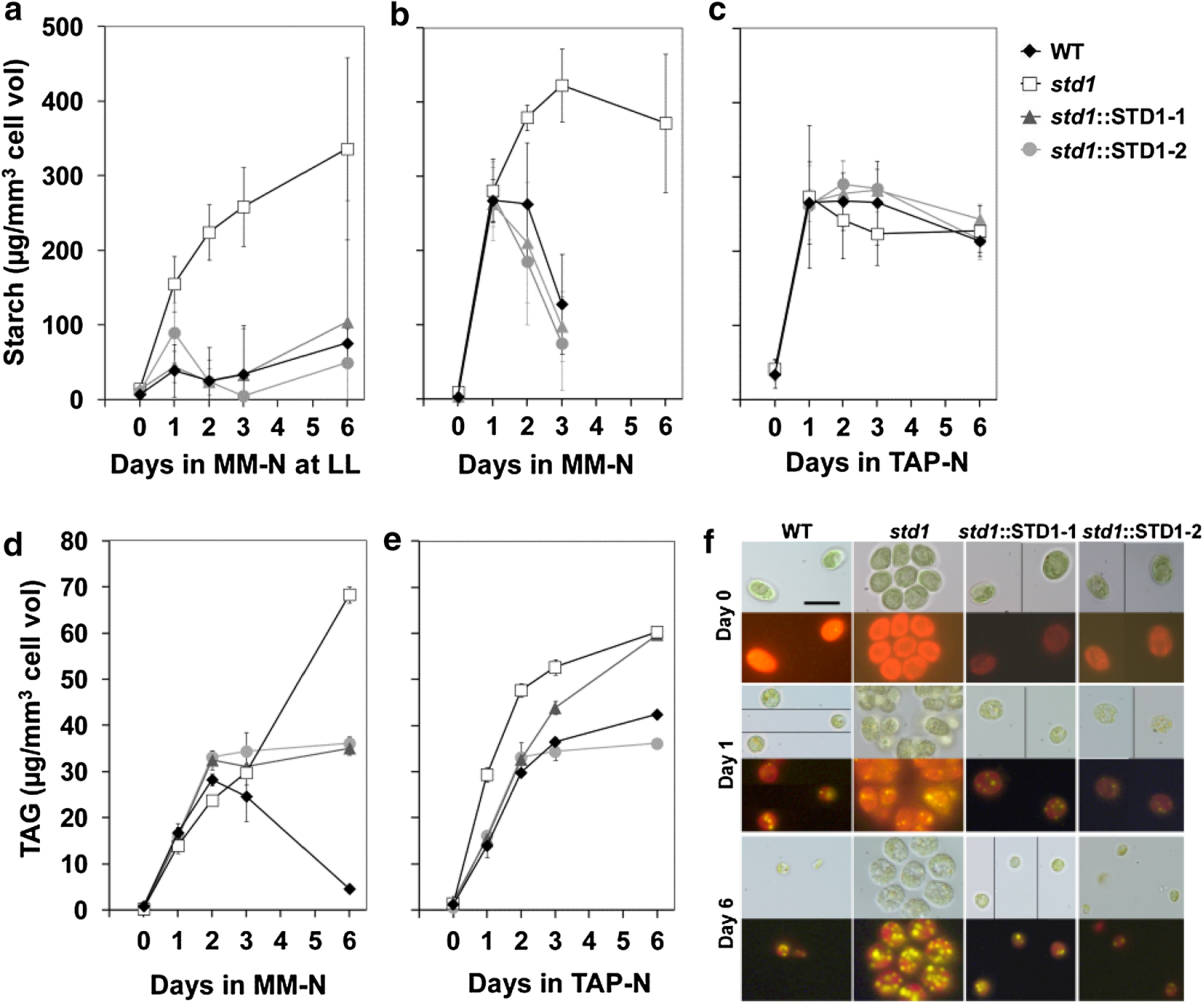
Starch and oil hyper-accumulate in *std1* mutant following N deprivation. **a** Starch content in cells grown photoautotrophically under low light (LL, 35 µmol photons m^−2^ s^−1^) supplemented with 2 % CO_2_. **b** Starch content in cells grown photoautotrophically under medium light (100 µmol photons m^−2^ s^−1^) supplemented with 2 % CO_2_. **c** Starch content in cells grown mixotrophically (i.e., TAP) under medium light (100 µmol photons m^−2^ s^−1^). **d** TAG content in cells grown photoautotrophically under medium light (100 µmol photons m^−2^ s^−1^) supplemented with 2 % CO_2_. **e** TAG content in cells grown mixotrophically (i.e., TAP media) under medium light (100 µmol photons m^−2^ s^−1^). **f** *Nile red* fluorescence (*lower panel*) and bright field microscopy (*upper panel*) of indicated cells under photoautotrophic N deprivation. *Scale bar* 10 µm. *MM* minimal medium. Data are means ± SD (*n* = 3). Cell counts, cellular volumes, and chlorophyll content measurements related to these experiments are shown on Additional file 1: Figure S4

Although *std1* was initially identified as a mutant affected in starch degradation [19] (Additional file 1: Figure S1C), further characterization identified an even stronger starch accumulation phenotype in response to depletion of nitrogen (Fig. 2a, b) or sulfur (Additional file 1: Figure S3). Interestingly, this phenotype was dependent on culture conditions. In WT and complemented strains, starch accumulation strongly depended on the intracellular energy status. Low or transient accumulations were respectively observed at low (35 µmol photons m^−2^ s^−1^) or medium light (100 µmol photons m^−2^ s^−1^) under photoautotrophic N deprivation (Fig. 2a, b), while high and persistent accumulation was observed in mixotrophic conditions, in which acetate was added to the culture medium (Fig. 2c). In the wild-type strain, accumulation of reserve compounds depends on light intensity or acetate supply, thus reflecting the effect of the intracellular energy status on reserve formation. This dependency was absent in *std1*, in which high and persistent starch accumulation was observed under all of these conditions, the wild-type phenotype being rescued in complemented strains (Fig. 2a–c). The intracellular oil content increase was also greater in the mutant than in control strains (Fig. 2f), although the difference occurred later during starvation in photoautotrophy (Fig. 2d) as compared to mixotrophy (Fig. 2e). While both complemented lines showed an intermediary oil phenotype on Fig. 2d, full restoration was observed in an independent experiment (Additional file 1: Figure S5).

### The *std1* mutant shows increased biomass production during photoautotrophic N starvation

**Fig. 3:**
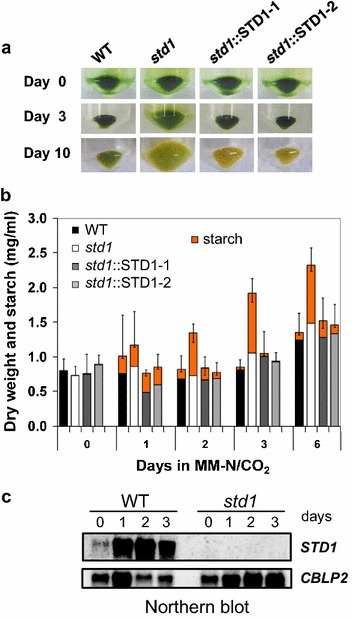
Biomass and starch production during photoautotrophic N deprivation. All strains were grown photoautotrophically in an MM medium supplemented with 2 % CO_2_, at a light intensity of 100 µmol photons m^−2^ s^−1^ and then subjected to N deprivation (at day 0). **a** Visual observation of cell pellets from 1 ml N-starved cells harvested at different time points. **b** Biomass (measured as dry weight) and intracellular starch were determined in N-starved strains. Data represent means ± SD (*n* = 3) (*upper error bar* dry weight, *lower error bar*, starch). **c** Northern blot analysis of *DYRKP* transcripts in response to N deprivation

Biomass production (as estimated from cell pellet size) was much higher in *std1* cultures than in control strains upon 3 and 10 days of N deprivation (Fig. 3a). While control lines grew as isolated cells, the *std1* mutant formed aggregates (or palmelloids [23]) of 2, 4, 8 or more cells enclosed by the mother cell wall (Additional file 1: Figure S6). Nonetheless, the cell number as determined after autolysin treatment increased in a similar manner in both *std1* and control strains; the large increase in the cellular volume observed in the mutant resulted from an increase in the volume of each single cell (Additional file 1: Figure S6). After 6 days of N depletion, the biomass increase (measured as dry weight) was about twice higher in the mutant than in control strains, the difference being essentially due to the increase in intracellular starch (Fig. 3b). Transcript analysis demonstrates that the *DYRKP* gene is strongly expressed after 1 day of N depletion, and remained high after 3 days of deprivation (Fig. 3c), thus indicating a functional role of the kinase in the response to nitrogen stress.

**Fig. 4:**
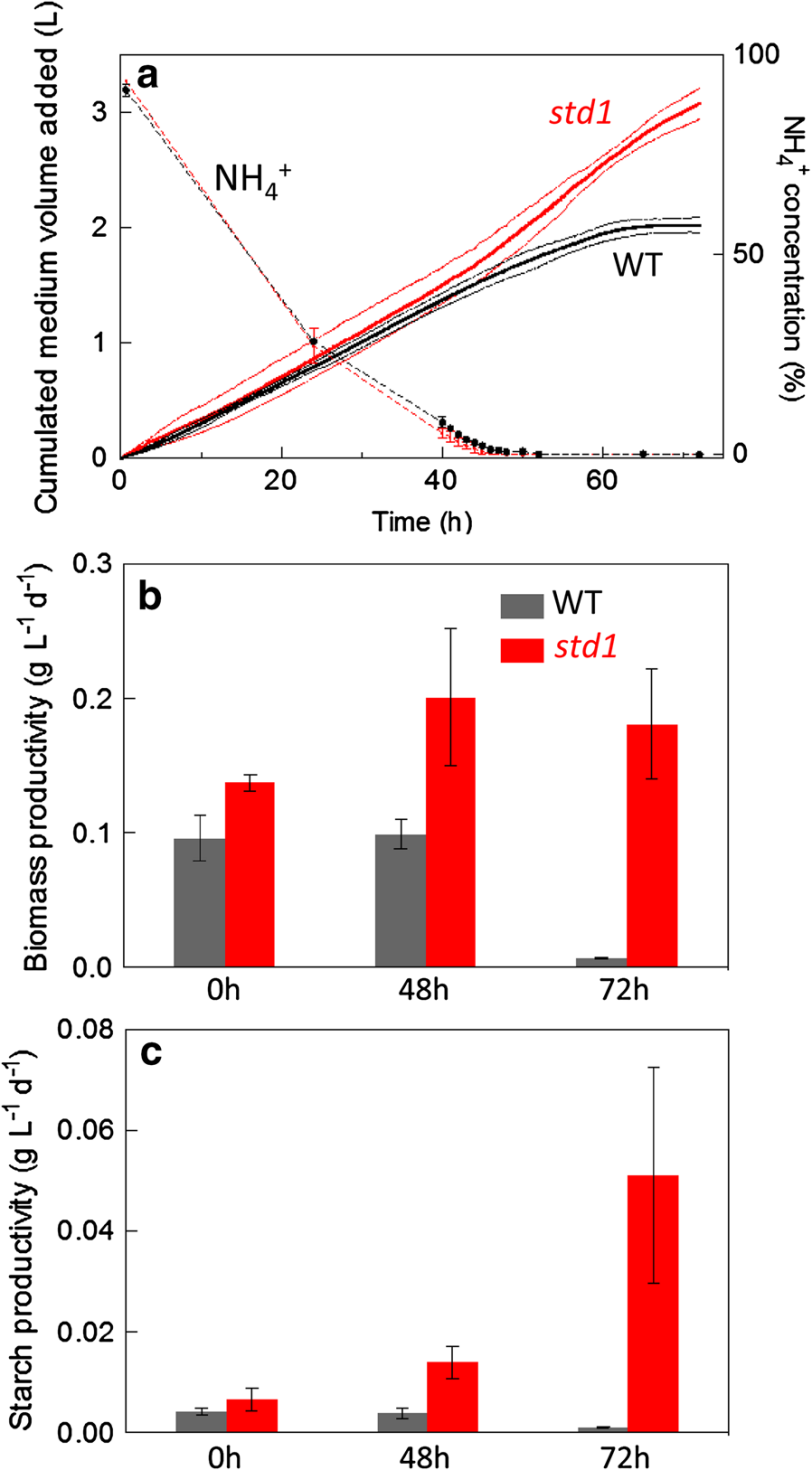
Biomass productivity of WT and *std1* *Chlamydomonas* cells measured in photobioreactors operated as turbidostats during photoautotrophic N deprivation. Cells were grown under constant illumination (125 µmol photons m^−2^ s^−1^) in the presence of 2 % CO_2_ enriched air. Cell density was measured using an absorption probe and maintained at a constant level by injection of fresh medium. Due to the aggregation phenotype of *std1*, OD_880nm_ was regulated at different values for WT (OD_880nm_ = 0. 4) and *std1* (OD_880nm_ = 0. 3) to reach similar biomass concentrations (0. 15 g dry weight L^−1^). After a 48-h stabilization period in the presence of MM, the dilution medium was replaced by MM-N (*t*_0_). **a** Cumulated amounts of fresh medium were added to maintain the culture at a constant biomass concentration. Measurements of ammonium concentration (*dotted lines*) in the culture medium showed complete exhaustion after 45 h. The 100 % value corresponds to 7.5 mM NH_4_^+^, which is the ammonium concentration of the minimal medium. Shown are mean ± SD (*n* = 3). **b** Biomass productivity (g dry weight L^−1^ d^−1^) was determined from dilution rates and biomass measurements at *t*_0_, and 48 and 72 h after N-removal. Shown are means ± SD (*n* = 3)

Biomass productivity was then measured by using photobioreactors operated as turbidostats (Fig. 4). WT and *std1* mutant cells were first grown under N replete conditions by maintaining biomass at a constant value through the addition of fresh MM. At t_0_, MM was replaced by N-deprived MM as dilution media, resulting in a complete N depletion in both cultures after 43 h (Fig. 4a). Upon starvation, the dilution rate of the *std1* culture increased while the dilution rate of the WT culture progressively decreased. Biomass productivity, determined from dilution rate and dry weight measurements, was much higher in the mutant after 48 h and remained at a high value after 72 h, while the productivity of the WT strongly decreased and reached a null value after 72 h (Fig. 4b). Starch productivity was also higher in the *std1* mutant than in the WT, the effect being more marked at 72 h (Fig. 4c).

### The *std1* mutant maintains higher photosynthetic activity under photoautotrophic N starvation

**Fig. 5:**
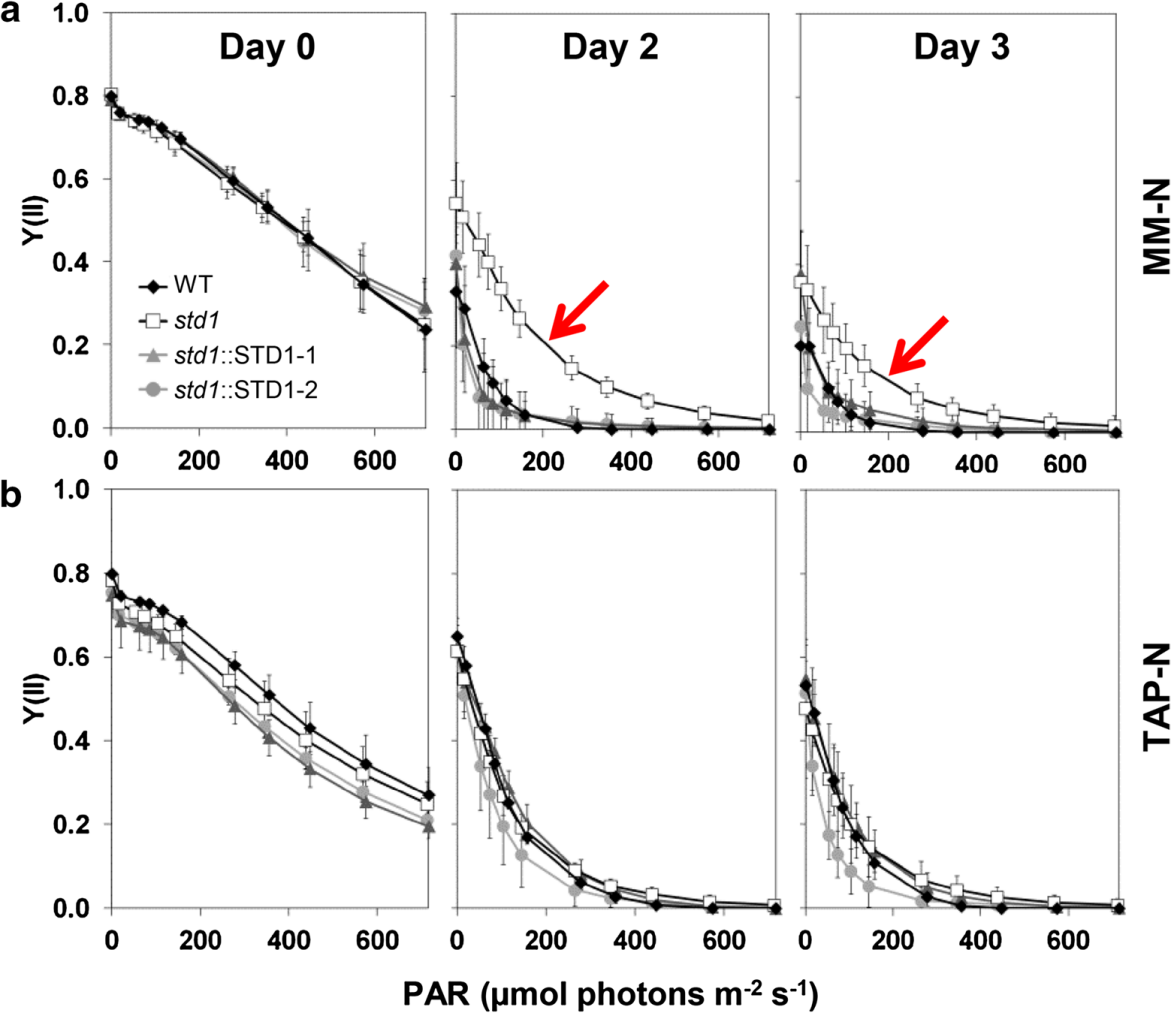
Photosynthetic activity of WT and *std1* *Chlamydomonas* cells during mixotrophic or photoautotrophic N deprivation. PSII yields were determined at different light intensities in the *std1* mutant, in the WT and in two complemented mutant lines by measuring pulse amplitude-modulated chlorophyll fluorescence. Cells were grown under a light intensity of 100 µmol photons m^−2^ s^−1^ under **a** Photoautotrophic conditions (i.e., MM supplemented with 2 % CO_2_) or **b** Mixotrophic conditions (i.e., TAP). At *t*_0_, cells were resuspended in an N-free medium and fluorescence measurements were performed at t_0_ (N-replete) and after 2 and 3 days of N deprivation. Data are means ± SD (*n* = 6 for **a**, and *n* = 4 for **b**)

N deprivation is known to induce a decline in the photosynthetic activity of microalgae, resulting in a drop in the PSII yield [9]. When N deprivation was performed under photoautotrophic conditions, the PSII yield decrease was less pronounced in *std1* than in control strains (Fig. 5a). However, parallel decreases in PSII yields were observed in *std1* and control strains when N deprivation was conducted under mixotrophic conditions (Fig. 5b). Immunodetection experiments revealed a decrease in major photosynthetic components in both *std1* and the WT progenitor in response to N deprivation (Additional file 1: Figure S7), therefore indicating that the higher photosynthetic activity observed in *std1* does not result from higher levels of photosynthetic complexes but rather from a higher PSII activity. Consistently, the higher photosynthetic activity of the mutant was observed in conditions where the synthesis of reserve compounds was increased (Fig. 2).

## Discussion

Accumulation of reserve compounds (starch and oil) by photosynthetic organisms is part of an acclimation strategy to nutrient shortage. Despite a strong interest in microalgae as a renewable feedstock for the production of next-generation biofuels, little is known about molecular mechanisms regulating carbon storage. Using the unicellular green alga *C. reinhardtii* as a model, we developed a forward genetic screen to identify novel genes involved in the regulation of starch dynamics [19]. We report here the characterization of a mutant (*std1*) affected in a DYRK kinase homologue belonging to a subgroup (called *DYRKP*) specific to plants. The *std1* mutant, the first DYRKP mutant reported so far, accumulates high intracellular starch and oil amounts and shows a sustained photosynthetic activity in response to nutrient starvation.

### DYRKP, a negative regulator of carbon storage

**Fig. 6:**
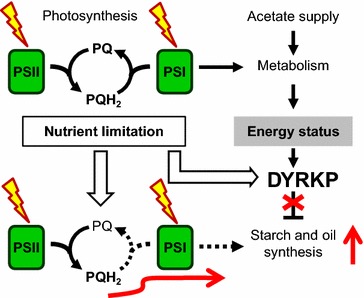
A hypothetical model of the DYRKP (STD1) function in response to nutrient deprivation. We propose that DYRKP negatively regulates the sink capacity in response to both nutrient and energy signals. DYRKP is induced in response to nutrient limitation, and would be active in conditions of low energy status. Disruption of *DYRKP* in *std1* allows sustained synthesis of reserve compounds, thereby increasing electron sink capacity and maintaining a high photosynthetic rate

In nutrient-deprived WT cells, accumulation of starch is strongly dependent on the cellular energy status. While low starch accumulation occurs at low light, higher accumulation is observed as light increases or acetate is supplied to the culture medium (Fig. 2a–c). This dependency is abolished in the *std1* mutant, in which high and sustained starch accumulation was observed in all conditions (Fig. 2a–c). We therefore suggest that the DYRKP (STD1) kinase acts as a negative regulator of carbon storage in conditions of low cellular energy status (Fig. 6). Under nutrient deprivation, photosynthesis would be restricted in the wild-type strain by the sink capacity of reserve metabolism, resulting in an increase in excitation pressure at PSII. This restriction would be alleviated in the *std1* mutant, thus explaining the lower excitation pressure and the higher photosynthetic activity observed in the mutant (Fig. 5a, b). In WT, the function of DYRKP (STD1) would be to prevent excessive accumulation of reserve compounds when the cellular energy status is low, thereby preserving cellular energy for other purposes. External acetate supply, which increases the intracellular energy status [24] has been reported to boost accumulation of reserves under nitrogen depletion [25, 26]. Such a stimulation of reserve accumulation by acetate, which is absent in *std1*, reflects the tight regulation of this phenomenon by the intracellular energy status, which likely occurs through a DYRKP (STD1)-dependent mechanism.

### DYRKP are plant-specific DYRK kinases

Phylogenetic analysis revealed that DYRKs can be divided into four subgroups, the DYRK1, the DYRK2, the Yak, and the DYRKP subgroup defined in this study (Fig. 2; Additional file 1: Figure S2, S8, S9). While vascular plant DYRKs only belong to two subgroups (Yak and DYRKP), algal and moss genomes also harbor members of the DYRK2 subgroup. Vascular plants and mosses possess more than one homologue of the DYRKP subgroup (*Arabidopsis* and *Populus* contain four members and *Physcomitrella* three) and of the Yak subgroup (*Physcomitrella* harbors five and *Arabidopsis* and rice have one and two, respectively). In contrast, algal genomes contain only one member of each DYRK subgroup (Yak, DYRK2, and DYRKP). Such a lower redundancy of algal genomes was previously reported for other gene families. For example, the *Chlamydomonas* genome harbors a smaller number of cell cycle genes than vascular plants or vertebrates [27]. Only two genes encoding heat-shock factors are found in the *Chlamydomonas* genome, whereas *Arabidopsis* has more than 21 [28]. While mutants of DYRK homologs of Yak1 have been recently isolated in *C. reinhardtii* [29] and *A. thaliana* [30], this is the first report on the function of a DYRKP kinase in the green lineage.

### DYRK functions and mechanisms

Yeast and mammalian DYRKs contain a conserved YxY motif in the so-called activation loop (subdomain VIII), which is autophosphorylated at the second tyrosine thus providing full activity of DYRK kinases [31, 32]. The autophosphorylation was described as a one-off event, which is mediated by an intramolecular mechanism and a transitory intermediate structure [33, 34]. The YxY motif is not conserved in the DYRKP group, the first tyrosine being replaced by a cysteine in plants and a serine in *C. reinhardtii* (Additional file 1: Figure S2A). However, for all DYRKs analyzed so far, only the second tyrosine of the YxY motif is phosphorylated and essential for the kinase activity [35]. Moreover, recent phosphoproteome studies have shown the existence of phospho-tyrosine peptides in the activation loops of *Arabidopsis* AtYak1 and AtDYRKP3 [36] and of *C. reinhardtii* CreYak1 and CreDYRKP [37]. Taken together, this strongly suggests that activation of plant and algal DYRKP kinases also depends on tyrosine autophosphorylation. In yeast, the DYRK homologue Yak1 has been reported to be part of a glucose-sensing system controlling the arrest of the cell cycle after translocation to the nucleus and by phosphorylating Pop2p [21]. Yak1 was also suggested to be involved in the control of glycogen storage [38] and to mediate stress responses by phosphorylation of transcription factors such as Msn2 and Hsf1 upon glucose depletion [39]. More recently, Yak1 was proposed to lie at the center of a regulatory cascade controlling growth and stress response by targeting different transcription factors [40]. Further studies will be needed to identify DYRKP targets and the nature of the regulatory cascade controlled by DYRKP in plants and microalgae.

### Relationships with other signaling pathways of nutrient and energy signals

In yeast and mammals, target of rapamycin (TOR) and RAS/cAMP are the major signaling pathways involved in the regulation of cell growth in response to nutrient and energy-derived signals [41]. The mTORC1 complex senses the energy status of a cell through AMP-activated protein kinase (AMPK) [42], and controls anabolic processes such as ribosome biogenesis and translation through the phosphorylation of different targets [42]. In photosynthetic eukaryotes, both SnRK1 (the plant orthologue of AMPK) and TOR are involved in the control of cellular processes through the transduction of nutrient and energy signals [12, 43]. In yeast, TOR negatively regulates the DYRK kinase Yak1 via PKA (cAMP-dependent protein kinase) [44]. Whereas the TOR pathway is conserved in all eukaryotes and controls cell proliferation and growth in response to nutrient and energy signals, DYRKP kinases are plant specific. We suggest here that in green algae and most likely in vascular plants, DYRKP is part of a regulatory cascade controlling the accumulation of reserve compounds, a key feature for the survival of photoautotrophic eukaryotes in fluctuating conditions of nutrient availability.

### DYRKP as a target for improving carbon storage in microalgae and plant crops

Increasing the accumulation of reserve compounds is a major issue for domestication of microalgae [1–4]. While great improvements of oil or starch content have been obtained in crop plants by means of classical breeding techniques, successful biotechnological improvements of microalgae are still scarce. Regulators of the algal oil content such as NRR1 [16] may represent potential targets for biotechnological improvements. It has been recently suggested that CHT7, a repressor of cellular quiescence could provide a target to increase biomass and oil productivity in algae [45]. Moreover, it has been hypothesized that vascular plants may increase the carbon flow into starch and other storage compounds by avoiding feedback inhibition of photosynthesis, for instance by the manipulation of signaling pathways mediated by SnRK protein kinases [13]. Such an effect was clearly observed here in the *std1* mutant, inhibition of the DYRKP kinase increases the storage of reserve compounds and the sink strength, thus limiting feedback inhibition of photosynthesis (Fig. 6). The discovery of DYRKP thus opens new perspectives for bioengineering of microalgae.

## Conclusions

Despite a strong interest for microalgal biofuels, little is known concerning regulatory mechanisms controlling cellular accumulation of storage compounds. By isolating a *Chlamydomonas* kinase mutant hyper-accumulating starch and oil in response to nitrogen starvation, we show here that accumulation of reserve compounds is a highly regulated process. The newly discovered DYRKP kinase is proposed to down-regulate reserve accumulation in the wild-type by integrating nutrient and energy signals, such a regulation being suppressed in the mutant. Future work will aim at identifying molecular targets of the kinase as well as the full signaling pathway involved in this regulatory mechanism. The DYRKP kinase therefore represents a valuable target for the biotechnological improvement of microalgae and other crops to enhance their energy density for the production of biofuel.

## Methods

### Strains and growth conditions

The *C. reinhardtii* wild-type strain CC124 (*mt−nit1 nit2*) was used for mutant generation as described previously [19]. The mutant strain *std1* was isolated from a mutant library generated by transformation of the strain CC124 with the *Kpn*I-linearized plasmid (pSL-X) harboring the paromomycin-resistance cassette *Aph*VIII [19]. The starch content of mutant strains was assayed using coloration with iodine vapor following 5 days of nitrogen starvation to trigger starch accumulation and 48 h in the dark on a nitrogen replete medium of starch to induce starch breakdown [19]. Cells were grown in flasks either mixotrophically (Tris–acetate–phosphate or TAP medium) [23] or photoautotrophically (MOPS-buffered minimal medium [23] supplemented with 2 % CO_2_ in the air), under continuous illumination (100 µmol photons m^−2^ s^−1^ or ~35 µmol photons m^−2^ s^−1^ for low-light experiment) at 25 °C. Precultures were grown to a density of 2 to 4 × 10^6^ cells ml^−1^ before starvation experiments. Due to palmelloid formation in *std1*, total cellular volume was measured using a Multisizer 3 Coulter counter (Beckman Coulter) and the different strains were diluted to reach a similar cellular concentration before starvation experiments.

Photobioreactor experiments were performed using four photobioreactors operated as turbidostats as described previously [46]. Cells were grown photoautotrophically on a minimal medium by bubbling air supplemented with 2 % CO_2_ under a light intensity of 125 µmol photons m^−2^ s^−1^. The pH was maintained at 7.2 by addition of 0.2 M KOH. At t_0_, the dilution medium was replaced by a nitrogen deprived minimal medium. Ammonium concentration was determined in the culture supernatant at different time points by using an ion selective electrode (Orion 9512HPBNWP, Thermo Scientific).

### Genetic characterization and complementation of the *std1* mutant

To check the integration frequency of the inserted DNA, Southern blot analysis was performed with wild-type and *std1* mutant cells. Genomic DNA was prepared as described previously [47], and 4, 6 or 8 µg genomic DNA restricted with *Not*I were separated in an 0.8 % agarose gel, blotted on a nylon membrane and hybridized with a digoxygenin-labeled probe complementary to part of the *Aph*VIII gene of the inserted resistance cassette. A PCR DIG Probe Synthesis Kit (Roche) was utilized for probe labeling using primers AphORF_For and Aph_Tail3. The hybridization with the resulting 400 bp-PCR fragment was performed overnight at 50 °C using DIG Easy Hyb buffer (Roche). Anti-Digoxigenin-AP and CSPD as substrate (Roche) were applied to detect signals using G:BOX Chemin XL (Syngene). The integration site of the paromomycin resistance cassette was identified by genome walking (GenomeWalker Kit, Clontech). Genomic DNA of the strain *std1* was digested with *Fsp*I and processed according to the manufacturer’s instructions. The gene-specific primers GSP1 and GSP2 allowed the determination of the genomic sequence downstream of the inserted *Aph*VIII cassette. Amplification was achieved using the Advantage GC Genomic LA Polymerase (Clontech). A ~1900 bp fragment from the ~4800 bp pSL-X was found inserted into the *std1* genome. For complementation of the *std1* mutant, the genomic DNA encoding *DYRKP* gene was amplified using the primers XbaG4forHyg and XbaG4RevHyg and DyNAzyme™ EXT DNA Polymerase (Finnzymes Oy). The amplified PCR product (6913 bp) was restricted by *Xba*I and cloned into the *Xba*I-digested vector pSL-Hyg (derived from pSL18 [48]), which is under control of the *psaD* promoter and carries a resistance cassette for hygromycin [49]. Following autolysin treatment, *std1* cells were transformed with *Kpn*I-linearized pSL-Hyg-STD1 by agitation with glass beads [50]. Transformed cells were selected on TAP plates containing 10 µg ml^−1^ hygromycin B and complemented lines were screened by applying the same protocol used for mutant isolation [19].

### Phylogenetic analysis

Amino acid sequences were aligned using MAFFT version 6 software [51]. The resulting alignment was then manually refined using SeaView version 4 [52]; regions displaying dubious homology were removed from further analysis. A total of 313 amino acid positions were retained for the phylogenetic analysis of DYRK proteins. Phylogenetic analyses were conducted using Neighbour-Joining (NJ), Maximum Likelihood (ML) and Parsimony (Pars) approaches in the Phylogenetic Inference Package Phylip (version 3.69) (Fig. 1d; Additional file 1: Figures S8, S9) [53]. The PROTML program was used for ML analysis and the sequence input order was randomized (20 jumbles). The SEQBOOT and CONSENSE programs were used for bootstrap value calculations on 100 replications and consensus tree reconstructions, respectively. To examine node confidence, NJ and Pars analyses were done by using the NEIGHBOR and PROTPARS programs. Distance matrices used for the NJ analysis were created with the PROTDIST program. The phylogenetic trees were drawn with MEGA5 [54].

### RNA analyses and RT-PCR

Total RNA was isolated as previously described [55]. For RT-PCR reactions, 1 µg of DNaseI-treated total RNA was utilized with the OneStep RT-PCR Kit (Qiagen). To obtain sequence information of the complete transcribed *DYRKP* (*STD1*) gene, three overlapping RT-PCRs were performed using the primer pairs Std1UTR1 and Std1P3rev, Std1FW2 and G4rev14, and the primer pair ACG4_FW3 and ACG4_Rev1. For comparison of transcript levels in wild-type, mutant and complemented strains, the Std1FW2–G4rev14 primer pair was used to amplify part of the *DYRKP* transcript. Specific primers were designed for actin (locus name Cre13.g603700, protein ID 515031) serving as a constitutively expressed control gene (Actin_FW and Actin_Rev).

For Northern blot analysis, RNA extraction was performed by collecting 15 ml of cell cultures at different time points on ice and centrifuging for 1 min at 1789*g*. The 500 µl cell suspension was then transferred to a 1.5 ml-tube on ice and mixed with 500 µl of RNA lysis buffer. RNA extraction, separation on formaldehyde agarose gels and northern blot probing were performed as previously described [55]. Membranes were hybridized with DNA probes containing a fragment of *STD1* or *CBLP2* as a loading control. A 1.1-kb *Bam*HI-*Hind*III fragment coding for the 3′-part of *DYRKP* (*STD1*) and the 1-kb cDNA of *CBLP2* were used for hybridization. The ACG4_FW3 and ACG4_Rev1 primers were used for RT-PCR, and the amplified product was restriction digested by *Bam*HI and *Hind*III, which was then cloned into the *Bam*HI–*Hind*III-restricted pQE-30 vector (Qiagen). Radioactive signals were detected using BAS-IP MS2040 phosphorimager plates (Raytest; http://www.raytest.de), scanned with a Molecular Imager FX phosphorimager (Bio-Rad; http://www.biorad.com), and imaged using the Quantity One-4.5.1 program (Bio-Rad).

### Protein extraction and immunoblot analysis

To detect DYRKP, soluble cell lysates were prepared as follows: 100 ml of *C. reinhardtii* cell culture in the exponential phase (eq. to 5 × 10^6^ cells ml^−1^ or 0.8 mm^3^ ml^−1^) were harvested by centrifugation for 2 min at 1789*g* and resuspended in 1 ml lysis buffer (20 mM HEPES–KOH pH 7.2, 10 mM KCl, 1 mM MgCl_2_, 154 mM NaCl, 0.1× protease inhibitor cocktail; Sigma P9599). Cells were sonicated on ice for 90 s with an alternating cycle of 1 s pulse/1 s pause. Lysates were loaded onto sucrose cushions and centrifuged in a MLA-55 rotor (Beckman Coulter) for 30 min at 151,300*g* and 4 °C. Soluble proteins were mixed with one volume of 2 × sample buffer [28] or 2 × LDS sample buffer (Invitrogen) and heated for 5 min at 95 °C or 10 min at 70 °C prior to loading on an 8 % SDS–polyacrylamide gel. Immunodetection of DYRKP (STD1) was performed using a purified antibody obtained by immunizing two rabbits against two synthetic peptides (DGMDDPGYSRKEVPNP-cys and PAVNHEDVELFRN-cys) conjugated to KLH (keyhole limpet hemocyanin) as the carrier protein (http://www.proteogenix-antibody.com/). DYRKP was detected by ECL (SuperSignal West Pico Chemiluminescent Substrate, Thermo Scientific). For immunodetection of other proteins, cell pellets equivalent to 1.2 mm^3^ total cellular volume were harvested from cell cultures and stored at −80 °C until use. Cell pellets were resuspended in 70 µl of a buffer containing 50 mM Tris pH 8, 10 mM EDTA and 2 % SDS, incubated for 30 min at RT, and centrifuged for 2 min at 4 °C. Protein concentrations were quantified from 2 µl samples by colorimetric measurements with bicinchonic acid (Pierce BCA Protein Assay kit, Thermo Scientific). For immunoblot analysis, 10–12 µg of total protein extracts were separated on 10 % SDS–polyacrylamide gels, transferred to BioTrace™ NT nitrocellulose membrane (Pall Life Sciences, http://www.pall.com) and decorated using antibodies raised against AtpB, COXIIb, Cyt f, PsaC, PsbD (D2), and RbcL (all purchased from Agrisera).

### Oil content quantification

*Chlamydomonas reinhardtii* cells (eq. to 2 mm^3^ total cellular volume) were harvested by centrifugation at 1000*g* for 2 min (at 4 °C). The cells were quenched in hot isopropanol for immediate lipid extractions. Total cellular lipids were extracted using a mixture of hexane and isopropanol [56]. Organic solvent phase-containing lipids were collected and dried under a stream of nitrogen gas, then resuspended into 200 µl chloroform:methanol (2:1, v/v). Triacylglycerols (TAGs, i.e., oils) were first separated from other lipid classes on a thin layer chromatograph and charred with 2 % CuSO_4_ dissolved in 8 % H_3_PO_4_ in water. TAG content was then calculated based on a densitometry method, after comparison to a standard curve generated with a C17:0 TAG standard [6].

### Chlorophyll fluorescence

Chlorophyll fluorescence was measured using a Dual Pam-100 (Heinz Walz). Samples were placed into a cuvette under constant stirring at room temperature and were dark-adapted for 5–10 min before measurements. Light curves were recorded by increasing stepwise (3 min per step) the light intensity from 15 to 715 µmol photons m^−2^ s^−1^. Saturating flashes (10,000 µmol photons m^−2^ s^−1^, 200 ms duration) were applied to determine PSII yield, 1-qP, and ETRs [57, 58].

### Biomass, starch, and chlorophyll measurements

Biomass was determined by dry weight measurements from three 5-ml samples upon filtration of the algal culture on a glass fiber filter (VWR, Ref. 611-0739) dried overnight at 80 °C. Intracellular starch and chlorophyll contents were measured as previously described [19].

### Microscopy

A Leica DMRXA microscope (Leica Microsystems, Germany) was used for light microscopy. When necessary, cells were fixed with 0.25 % glutaraldehyde in the medium. A Neubauer chamber was used to compare cell concentrations. Images were captured with the Spot Insight 4 software (Diagnostic Instruments Inc., Sterling Heights, USA). Fluorescence microscopy following Nile red staining was performed as previously described [6], but with the addition of DMSO (10 %, v/v) to aid in the penetration of Nile red through several cell wall layers of mutant cells.

## Additional file

10.1186/s13068-016-0469-2 Southern blot analysis and complementation of the std1 mutant.** Figure S2.** Conserved sequence features of DYRKP kinases.** Figure S3.** Persistently high starch levels were observed in the std1 mutant during photoautotrophic S deprivation conditions.** Figure S4.** Cell counts, total cellular volume data and chlorophyll contents for the kinetic experiments in nitrogen deprivation shown on Fig. 2.** Figure S5.** Oil accumulates in std1 mutant following N deprivation.** Figure S6.** The std1 mutant forms cell aggregates enclosed by the mother cell wall.** Figure S7.** Protein levels in wild-type and std1 mutant cells during photoautotrophic N deprivation as determined by immunodetection.** Figure S8.** Phylogenetic tree of the DYRK protein family.** Figure S9.** Phylogenetic tree of the DYRK protein family by using the Maximum Likelihood (ML) or the Parsimony (Pars) approach.** Table S1**. Accession numbers of the sequences used for the phylogenetic tree in Fig. 1d.** Table S2.** List of primers used in this study.

## Abbreviations

DYRK: dual-specificity tyrosine-phosphorylation-regulated kinase
DYRKP: plant-specific dual-specificity tyrosine-phosphorylation-regulated kinase
MM: minimal medium
PSII: photosystem II
std1: starch degradation 1
TAP: tris–acetate–phosphate medium

## References

[1] Hu Q, Sommerfeld M, Jarvis E, Ghirardi M, Posewitz M, Seibert M (2008). Microalgal triacylglycerols as feedstocks for biofuel production: perspectives and advances. Plant J.

[2] Wijffels RH, Barbosa MJ (2010). An outlook on microalgal biofuels. Science.

[3] Georgianna DR, Mayfield SP (2012). Exploiting diversity and synthetic biology for the production of algal biofuels. Nature.

[4] Larkum AWD, Ross IL, Kruse O, Hankamer B (2012). Selection, breeding and engineering of microalgae for bioenergy and biofuel production. TiBiotech.

[5] Ball SG, Dirick L, Decq A, Martiat JC, Matagne RF (1990). Physiology of starch storage in the monocellular alga *Chlamydomonas reinhardtii*. Plant Sci.

[6] Siaut M, Cuine S, Cagnon C, Fessler B, Nguyen M, Carrier P (2011). Oil accumulation in the model green alga *Chlamydomonas reinhardtii*: characterization, variability between common laboratory strains and relationship with starch reserves. BMC Biotechnol..

[7] Delrue F, Li-Beisson Y, Setier PA, Sahut C, Roubaud A, Froment AK (2013). Comparison of various microalgae liquid biofuel production pathways based on energetic, economic and environmental criteria. Bioresource Technol.

[8] Merchant SS, Kropat J, Liu BS, Shaw J, Warakanont J (2012). TAG, You’re it! *Chlamydomonas* as a reference organism for understanding algal triacylglycerol accumulation. Curr Op Biotechnol.

[9] Peltier G, Schmidt GW (1991). Chlororespiration—an adaptation to nitrogen deficiency in *Chlamydomonas reinhardtii*. Proc Natl Acad Sci USA.

[10] Grossman A (2000). Acclimation of *Chlamydomonas reinhardtii* to its nutrient environment. Protist.

[11] Wykoff DD, Davies JP, Melis A, Grossman AR (1998). The regulation of photosynthetic electron transport during nutrient deprivation in *Chlamydomonas reinhardtii*. Plant Physiol.

[12] Robaglia C, Thomas M, Meyer C (2012). Sensing nutrient and energy status by SnRK1 and TOR kinases. Curr Opin Plant Biol.

[13] Coello P, Hey SJ, Halford NG (2011). The sucrose non-fermenting-1-related (SnRK) family of protein kinases: potential for manipulation to improve stress tolerance and increase yield. J Exp Bot.

[14] Gonzalez-Ballester D, Pollock SV, Pootakham W, Grossman AR (2008). The central role of a SNRK2 kinase in sulfur deprivation responses. Plant Physiol.

[15] Sato A, Matsumura R, Hoshino N, Tsuzuki M, Sato N. Responsibility of regulatory gene expression and repressed protein synthesis for triacylglycerol accumulation on sulfur-starvation in *Chlamydomonas reinhardtii*. Front Plant Sci. 2014;5. 10.3389/fpls.2014.00444 PMC4160968 25309550

[16] Boyle NR, Page MD, Liu BS, Blaby IK, Casero D, Kropat J (2012). Three acyltransferases and nitrogen-responsive regulator are implicated in nitrogen starvation-induced triacylglycerol accumulation in *Chlamydomonas*. J Biol Chem.

[17] Aranda S, Laguna A, de la Luna S (2011). DYRK family of protein kinases: evolutionary relationships, biochemical properties, and functional roles. Faseb J.

[18] Kajikawa M, Sawaragi Y, Shinkawa H, Yamano T, Ando A, Kato M (2015). Algal dual-specificity tyrosine phosphorylation-regulated kinase, triacylglycerol accumulation regulator1, regulates accumulation of triacylglycerol in nitrogen or sulfur deficiency. Plant Physiol.

[19] Chochois V, Constans L, Dauvillée D, Beyly A, Solivérès M, Ball S (2010). Relationships between PSII-independent hydrogen bioproduction and starch metabolism as evidenced from isolation of starch catabolism mutants in the green alga *Chlamydomonas reinhardtii*. Int J Hydrogen Energ.

[20] Garrett S, Broach J (1989). Loss of Ras activity in *Saccharomyces cerevisiae* is suppressed by disruptions of a new kinase gene, *YAKI*, whose product may act downstram of the cAMP-dependent protein kinase. Genes Dev.

[21] Moriya H, Shimizu-Yoshida Y, Omori A, Iwashita S, Katoh M, Sakai A (2001). Yak1p, a DYRK family kinase, translocates to the nucleus and phosphorylates yeast Pop2p in response to a glucose signal. Genes Dev.

[22] Becker W, Joost HG (1999). Structural and functional characteristics of Dyrk, a novel subfamily of protein kinases with dual specificity. Prog Nucl Acid Res Mol Biol.

[23] Harris EH (2009). The *Chlamydomonas* sourcebook.

[24] Cournac L, Latouche G, Cerovic Z, Redding K, Ravenel J, Peltier G (2002). *In vivo* interactions between photosynthesis, mitorespiration, and chlororespiration in *Chlamydomonas reinhardtii*. Plant Physiol.

[25] Goodenough U, Blaby I, Casero D, Gallaher SD, Goodson C, Johnson S (2014). The path to triacylglyceride obesity in the *sta6* strain of *Chlamydomonas reinhardtii*. Eukaryot Cell.

[26] Goodson C, Roth R, Wang ZT, Goodenough U (2011). Structural correlates of cytoplasmic and chloroplast lipid body synthesis in *Chlamydomonas reinhardtii* and stimulation of lipid body production with acetate boost. Eukaryot Cell.

[27] Bisova K, Krylov DM, Umen JG (2005). Genome-wide annotation and expression profiling of cell cycle regulatory genes in C*hlamydomonas reinhardtii*. Plant Physiol.

[28] Schulz-Raffelt M, Lodha M, Schroda M (2007). Heat shock factor 1 is a key regulator of the stress response in *Chlamydomonas*. Plant J.

[29] Kajikawa M, Sawaragi Y, Shinkawa H, Yamano T, Ando A, Kato M (2015). Algal dual-specificity tyrosine phosphorylation-regulated kinase, triacylglycerol accumulation regulator1, regulates accumulation of triacylglycerol in nitrogen or sulfur deficiency. Plant Physiol..

[30] Kim D, Ntui VO, Zhang N, Xiong L (2015). Arabidopsis Yak1 protein (AtYak1) is a dual specificity protein kinase. FEBS Lett.

[31] Kassis S, Melhuish T, Annan RS, Chen SL, Lee JC, Livi GP (2000). *Saccharomyces cerevisiae* Yak1p protein kinase autophosphorylates on tyrosine residues and phosphorylates myelin basic protein on a C-terminal serine residue. Biochem J.

[32] Himpel S, Panzer P, Eirmbter K, Czajkowska H, Sayed M, Packman LC (2001). Identification of the autophosphorylation sites and characterization of their effects in the protein kinase DYRK1A. Biochem J.

[33] Lochhead PA, Sibbet G, Morrice N, Cleghon V (2005). Activation-loop autophosphorylation is mediated by a novel transitional intermediate form of DYRKs. Cell.

[34] Soundararajan M, Roos AK, Savitsky P, Filippakopoulos P, Kettenbach AN, Olsen JV (2013). Structures of Down syndrome kinases, DYRKs, reveal mechanisms of kinase activation and substrate recognition. Structure.

[35] Han JF, Miranda-Saavedra D, Luebbering N, Singh A, Sibbet G, Ferguson MAJ (2012). Deep evolutionary conservation of an intramolecular protein kinase activation mechanism. Plos ONE..

[36] Mithoe SC, Boersema PJ, Berke L, Snel B, Heck AJR, Menke FLH (2012). Targeted quantitative phosphoproteomics approach for the detection of phospho-tyrosine signaling in plants. J Proteome Res.

[37] Wang HX, Gau B, Slade WO, Juergens M, Li P, Hicks LM (2014). The global phosphoproteome of *Chlamydomonas reinhardtii* reveals complex organellar phosphorylation in the flagella and thylakoid membrane. Mol Cell Proteomics.

[38] Wilson WA, Roach PJ, Montero M, Baroja-Fernandez E, Munoz FJ, Eydallin G (2010). Regulation of glycogen metabolism in yeast and bacteria. FEMS Microbiol Rev.

[39] Lee P, Cho BR, Joo HS, Hahn JS (2008). Yeast Yak1 kinase, a bridge between PKA and stress-responsive transcription factors, Hsf1 and Msn2/Msn4. Mol Microbiol.

[40] Malcher M, Schladebeck S, Mosch HU (2011). The Yak1 protein kinase lies at the center of a regulatory cascade affecting adhesive growth and stress resistance in *Saccharomyces cerevisiae*. Genetics.

[41] Rohde JR, Bastidas R, Puria R, Cardenas ME (2008). Nutritional control via Tor signaling in *Saccharomyces cerevisiae*. Curr Opin Microbiol.

[42] Wullschleger S, Loewith R, Hall MN (2006). TOR signaling in growth and metabolism. Cell.

[43] Baena-Gonzalez E, Sheen J (2008). Convergent energy and stress signaling. TIPS.

[44] Martin DE, Soulard A, Hall MN (2004). TOR regulates ribosomal protein gene expression via PKA and the forkhead transcription factor FHL1. Cell.

[45] Tsai CH, Warakanont J, Takeuchi T, Sears BB, Moellering ER, Benning C (2014). The protein compromised hydrolysis of triacylglycerols 7 (CHT7) acts as a repressor of cellular quiescence in Chlamydomonas. Proc Natl Acad Sci USA.

[46] Dang K-V, Plet J, Tolleter D, Jokel M, Cuine S, Carrier P (2014). Combined increases in mitochondrial cooperation and oxygen photoreduction compensate for deficiency in cyclic electron flow in *Chlamydomonas reinhardtii*. Plant Cell.

[47] Tolleter D, Ghysels B, Alric J, Petroutsos D, Tolstygina I, Krawietz D (2011). Control of hydrogen photoproduction by the proton gradient generated by cyclic electron flow in *Chlamydomonas reinhardtii*. Plant Cell.

[48] Dauvillee D, Stampacchia O, Girard-Bascou J, Rochaix JD (2003). Tab2 is a novel conserved RNA binding protein required for translation of the chloroplast *psaB* mRNA. EMBO J.

[49] Berthold P, Schmitt R, Mages W (2002). An engineered *Streptomyces hygroscopicus aph 7″* gene mediates dominant resistance against hygromycin B in *Chlamydomonas reinhardtii*. Protist..

[50] Kindle KL (1990). High-frequency nuclear transformation of *Chlamydomonas reinhardtii*. Proc Natl Acad Sci USA.

[51] Katoh K, Misawa K, Kuma K, Miyata T (2002). MAFFT: a novel method for rapid multiple sequence alignment based on fast Fourier transform. Nucleic Acids Res.

[52] Gouy M, Guindon S, Gascuel O (2010). SeaView version 4: a multiplatform graphical user interface for sequence alignment and phylogenetic tree building. Mol Biol Evol.

[53] Felsenstein J (2005). PHYLIP—Phylogeny Inference Package (Version 3.2). Cladistics.

[54] Tamura K, Peterson D, Peterson N, Stecher G, Nei M, Kumar S (2011). MEGA5: molecular evolutionary genetics analysis using maximum likelihood, evolutionary distance, and maximum parsimony methods. Mol Biol Evol.

[55] Liu CM, Willmund F, Whitelegge JP, Hawat S, Knapp B, Lodha M (2005). J-domain protein CDJ2 and HSP70B are a plastidic chaperone pair that interacts with vesicle-inducing protein in plastids 1. Mol Biol Cell.

[56] Li-Beisson Y, Shorrosh B, Beisson F, Andersson MX, Arondel V, Bates PD (2010). Acyl-lipid metabolism. Arabidopsis Book.

[57] Schreiber U, Schliwa U, Bilger W (1986). Continuous recording of photochemical and nonphotochemical chlorophyll fluorescence quenching with a new type of modulation fluorometer. Photosynth Res.

[58] Klüghammer C, Schreiber U. Saturation pulse method for assessment of energy conversion in PS I. PAM application notes (PAN) http://www.walz.com. 2008;1:11–4.

