# Supplementary material for: Hyper-accumulation of starch and oil in a Chlamydomonas mutant affected in a plant-specific DYRK kinase
Source: Biotechnol Biofuels. 2016 Mar 8;9:55. doi: 10.1186/s13068-016-0469-2 (PMC4782384; doi:10.1186/s13068-016-0469-2)
Supplement: Supplementary file 1 — 10.1186/s13068-016-0469-2 Southern blot analysis and complementation of the std1 mutant. Figure S2. Conserved sequence features of DYRKP kinases. Figure S3. Persistently high starch levels were observed in the std1 mutant during photoautotrophic S deprivation conditions. Figure S4. Cell counts, total cellular volume data and chlorophyll contents for the kinetic experiments in nitrogen deprivation shown on Fig. 2. Figure S5. Oil accumulates in std1 mutant following N deprivation. Figure S6. The std1 mutant forms cell aggregates enclosed by the mother cell wall. Figure S7. Protein levels in wild-type and std1 mutant cells during photoautotrophic N deprivation as determined by immunodetection. Figure S8. Phylogenetic tree of the DYRK protein family. Figure S9. Phylogenetic tree of the DYRK protein family by using the Maximum Likelihood (ML) or the Parsimony (Pars) approach. Table S1. Accession numbers of the sequences used for the phylogenetic tree in Fig. 1d. Table S2. List of primers used in this study. [file 13068_2016_469_MOESM1_ESM.docx]

**Supplemental Figures**

**Figure S1.** **Southern blot analysis and** **complementation of the *std1* mutant.**

**(A)** Southern blot analysis indicates single insertion of the antibiotic resistance cassette in the genome of *std1*. *Not*I-, *Xma*I or *Stu*I/*Sac*I-restricted genomic DNA of wild-type and *std1* mutant cells was loaded on an agarose gel, Southern blotted and hybridized with a probe against the *Aph*VIII gene (paromomycin resistance cassette). Loaded amount of DNA per lane is indicated in µg.

**(B)** Vector construct used for the complementation of the *std1* mutant. Genomic wild-type DNA coding for *STD1* was amplified by PCR and cloned into the pSL-Hyg vector harbouring the *psaD* promoter and terminator, and a hygromycin resistance marker allowing selection of positive transformants.

**(C)** Starch degradation phenotype of the *std1* mutant and of two complemented lines. Intracellular starch levels of wild-type strain (black), *std1* mutant (white) and two complemented strains (*std1*::STD1-1 and -2, grey) were analyzed in response to nutrient deprivation and resupply. Cultures were grown in TAP medium (Con. = Control), subjected for two days to nitrogen (TAP-N) or sulfur starvation (TAP-S), which induced starch accumulation (Acc.). Subsequently, the starved cells were transferred to minimal medium (MM) and kept for 8 or 24 hours in the dark. Starch was catabolized (Degr.) in MM (comprising N but no C) in the dark. Starch values are the means ± SD (n ≥ 3).

**Figure S2. Conserved sequence features of DYRKP kinases.**

**(A)** Alignment of amino acid sequences from the C-terminal kinase domain of *Chlamydomonas* DYRKP, five higher plant DYRKP homologs and one DYRKP homolog from moss. The sequences were aligned using ClustalW and shaded using BoxShade (<http://www.ch.embnet.org>). Amino acids highlighted in black are perfectly conserved, and similar residues are indicated by a grey background. Arrows above the alignment indicate the DYRK homology (DH) box that precedes the conserved catalytic kinase domain and the kinase subdomains I-XI according to [Aranda et al., 2011](#_ENREF_1). Subdomains I and II respectively harbour the ATP anchor and the phosphate anchor. The catalytic loop is found within subdomain VIb, the cation binding site is located within subdomain VII, and subdomain VIII contains the activation loop and the P+1 loop. The conserved tyrosine in the activation loop (“YxY”) was found to be autophosphorylated and is marked by a triangle. The segment between subdomains X and XI of the protein is known as the CMGC insert. For details see [Aranda et al., 2011](#_ENREF_1).

**(B)** Consensus sequence of the DH-box of DYRKs according to **(A)**, additional file 1: Figure S8, Figure S9, and Table S1. Below the published DH consensus sequence from (Becker and Joost, 1999), consensus sequences of DYRK1 (7 sequences), DYRK2 (22 sequences), and Yak1 (21 sequences) are shown. Three minor subgroups were distinguished within the DYRKP subgroup, DYRKP-A (12 land plant sequences including moss), DYRKP-B (11 vascular plant sequences), DYRKP-algae (7 sequences). Multiple sequence alignments of DH-box motifs were performed using the program WebLogo (<http://weblogo.berkeley.edu/logo.cgi>).

**(C)** Schematic illustration of protein domain organization in two Yak1 and two DYRKP representatives from *C. reinhardtii* (CreYak1 and CreDYRKP) and *A. thaliana* (AtYak1 and AtDYRKP-1). DH, DYRK-homology box; Kinase, kinase domain.

**Figure S3. Persistently high starch levels were observed in the *std1* mutant during photoautotrophic S deprivation conditions.**

Intracellular starch was measured in wild-type (black), *std1* mutant (white), and complemented lines (grey) during photoautotrophic S deprivation under medium light (100 µmol photons m^-2^ s^-1^). Starch values are the means of 5 biological replicates ± SD.

**Figure S4. Total cellular volume data and chlorophyll contents for the kinetic experiments in nitrogen deprivation shown on Figure 2.**

**(A, B, C)** Total cellular volume measurements in µm^3^/ml were recorded by Multisizer™ 3 Coulter Counter® (Beckman). Cells were kept photoautotrophically at low light (LL, 35 µmol photons m^-2^ s^-1^) **(A)** or medium light (100 µmol photons m^-2^ s^-1^) **(B)** supplemented with 2% CO_2_ **(A, B)** or mixotrophically (TAP) under medium light **(C)** as in Figure 2. Values are means ± SD (n ≥ 3).

**(D, E, F)** Chlorophyll contents during N deprivation in photoautotrophic or mixotrophic conditions. Chlorophyll was extracted by methanol, and chlorophyll a and b were determined by measuring the absorbance at 653, 666 and 750 nm using UV-VIS spectrophotometer (SAFAS UVmc2 with the software SP2000). Chlorophyll content was calculated using the formula Concentration (µg/ml) = (A1-A3)*19.71+(A2-A3)*4.44. Chlorophyll concentrations are means ± SD of ≥ 3 experiments, duplicate samples were taken.

**(G, H, J)** Cell or particle concentrations per ml were recorded by Multisizer™ 3 Coulter Counter® (Beckman) as in **(A, B, C)**.

**Figure S5. Oil accumulate in *std1* mutant following N deprivation.**

TAG content in cells grown photoautotrophically under medium light (100 µmol photons m^-2^ s^-1^) supplemented with 2% CO_2_. A representative experiment is shown with means from three technical replicates.


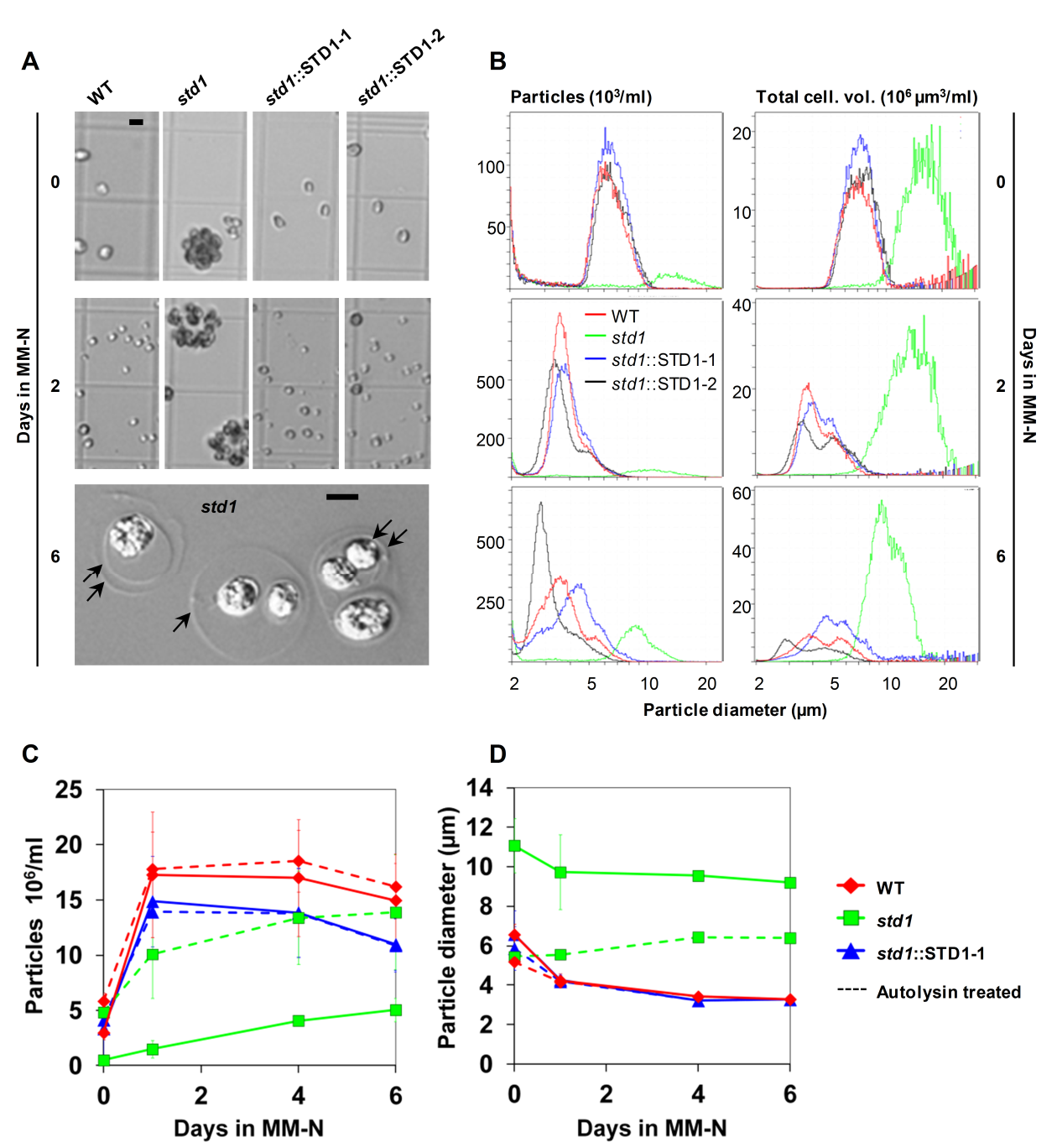


**Figure S6. The *std1* mutant forms cell aggregates enclosed by the mother cell wall.**

**(A)** Bright field and differential interference contrast images of wild-type (WT), *std1* mutant and complemented (*std1*::STD1-1 and -2) strains grown in minimal medium (MM) supplemented with 2% CO_2_ and then subjected to N deprivation for 0, 2 or 6 days. Arrows indicate mother cell walls. Scale bars, 10 µm.

**(B)** Particle numeration (left panels) and total cellular volumes (right panels) as a function of the particle diameter, determined in cultures of WT (red), *std1* mutant (green) and two complemented strains *std1*::STD1-1 (blue) and -2 (black) at 0, 2 or 6 days in MM-N/2% CO_2_. Data are from the same experiment as in **(A)**.

**(C, D)** Cell counting during nitrogen starvation following treatment with the cell wall-degrading enzyme autolysin. Particle concentration **(C)** and particle diameter **(D)** were determined in WT (red), *std1* mutant (green) and complemented lines (blue) during nitrogen deprivation in photoautotrophic condition (MM-N/2% CO_2_). Data are from before (solid lines) and after treatment with autolysin (dashed lines) and represent means ± SD (n = 4). Autolysin treatment allowed determination of *std1* cell number during deprivation kinetics **(C)** and revealed an increase in the volume of each single mutant cell, while the volume of wild-type cells decreased during nutrient starvation **(D)**.

**Figure S7.** **Protein levels in wild-type and *std1* mutant cells during photoautotrophic N deprivation as determined by immunodetection.**

**(A)** Provided antibody names include RbcL, Rubisco large subunit; Cyt f, cytochrome f; PsbD, D2 photosystem II subunit; PsaC, PSI-C core photosystem I subunit; AtpB, beta subunit of ATP synthase; and COXIIb, cytochrome oxidase subunit II b. 10 µg of total protein were loaded from N starved cells and harvested at the indicated time points after N depletion. Days in MM-N are indicated.

**(B)** Coomassie brilliant blue coloration. Molecular weight marker units are in kDa.

**Figure S8. Phylogenetic tree of the DYRK protein family.**

The complete tree obtained by the Neighbour-Joining (NJ) method as in Figure 1d, before combination of branches from *Populus trichocarpa*, *Zea mays*, and two species of *Micromonas* and *Ostreococcus*. Bootstrap values are indicated.

**Figure S9.** **Phylogenetic tree of the DYRK protein family by using the Maximum Likelihood (ML) or the Parsimony (Pars) approach.** Phylogenetic tree obtained by the ML **(A)** or the Pars **(B)** method. Branches were combined as displayed in Figure 1d.

**Table S1.** **Accession numbers of the sequences used for the phylogenetic tree in Figure 1D**.

Sequences in grey were not utilized for the alignment. Definitively incomplete gene models are indicated by an asterisk. When the predicted number of amino acids differed between two compared genome databases, the longer version was typically selected. Several genes display different splice variants, *e.g.* “ZmDYRKP3”, which harbours three transcripts at this locus. For *Danio rerio* and *Xenopus laevis*, not all existing *DYRK* genes were provided for the alignment. Data were obtained from NCBI and the following genome websites: <http://www.arabidopsis.org>, <http://rice.plantbiology.msu.edu>, <http://www.phytozome.org>, <http://www.maizesequence.org>, <http://www.cosmoss.org>, <http://genome.jgi.doe.gov/>.

**Table S1.** **Accession numbers of the sequences used for the phylogenetic tree in Figure 1D, continued.**

Alternative names of gene models: ^1^LOC_Os02g47410; ^2^LOC_Os04g51370; ^3^LOC_Os01g61620; ^4^LOC_Os03g51020, AAT77851, GI:50540694; ^5^LOC_Os05g39080, EEE64022, GI222631890.

**Table S1.** **Accession numbers of the sequences used for the phylogenetic tree in Figure 1D, continued.**

Predictions concerning the subgroup of incomplete gene models (indicated by an asterisk) from *Ostreococcus* and *Micromonas* species are difficult due to missing information about the N-terminal part including the DH-box.

**Table S2.** **List of primers used in this study.**

| **Name** | **Sequence** |
| --- | --- |
| AphORF_For | CGAAGCATGGACGATGCGTT |
| Aph_tail3 | CGAGACTGCGATCGAACGGACA |
| GSP1 | CTGGTGCTGCGCGAGCTGGCCCACGAGGAG |
| GSP2 | TGGTTCGGGCCGGAGTGTTCCGCGGCGTT |
| XbaG4forHyg | GTCTAGAATGTCGCTCCGCCTGAACCGATG |
| XbaG4RevHyg | GTCTAGACTACATGCTGTCGAGCGAGG |
| Std1UTR1 | CATAGTGCTCAGCAGGGGACAAGGC |
| Std1P3rev | AGCGTGCCAGAGGTTTCGCCGTC |
| Std1FW2 | CCGCGGACGGCGAAACCTCTGGCAC |
| G4rev14 | GATCTCGTCCAGCGACTGGTCAAAGTAG |
| ACG4_FW3 | GCGGATCCGACGAGCAGGGCAACGTGCTG |
| ACG4_Rev1 | CGGCAAGCTTCTACATGCTGTCGAGCGAGG |
| Actin_FW | AATCGTGCGCGACATCAAGGAGAA |
| Actin_Rev | TTGGCGATCCACATTTGCTGGAAGGT |

**Supplemental references**

Aranda, S., Laguna, A., and de la Luna, S. (2011). DYRK family of protein kinases: evolutionary relationships, biochemical properties, and functional roles. Faseb J 25, 449-462.

Becker, W., and Joost, H.G. (1999). Structural and functional characteristics of Dyrk, a novel subfamily of protein kinases with dual specificity. Prog. Nucl. Acid. Res. Mol. Biol. 62, 1-17.
